# Abstract of the Proceedings of the Buffalo Medical Association

**Published:** 1871-05

**Authors:** W. C. Phelps

**Affiliations:** Secretary; Buffalo


ART. III.—*Abstract of the Proceedings of the Buffalo Medical Association.*

BUFFALO, May 2d, 1871.

The President, Dr. JOHNSON, in the Chair.

Dr. STRONG was called, January last, to a case of croup, the patient being a girl three years of age. From the want of success in his previous treatment of this truly formidable disease, he had very little confidence in the efficacy of medicine; and, in common with the mass of the profession, had come to regard true croup as a fatal malady. The symptoms were very conclusive fever, some difficulty in breathing, slight cough during the first twenty-hours, all of which were more marked the next day, so that at night he had no hope of seeing his patient alive the next morning. The treatment had consisted mainly in the exhibition of quinia and calomel; but that night, rather as a last resort, ordered the inhalation of vapor in a room kept at a high temperature, about 90°, and also ordered bicarbonate of soda to be dissolved in the water which was evaporated. These instructions were strictly carried out, aud the next morning found the patient greatly improved and in a fair way to recover. Tonics were afterwards administered, and the patient was well a few days afterwards. The most convenient method of generating the vapor was to place the patient on a chair and throw over him a quilt, then place a pail, containing the hot water, in which the soda has been dissolved, under the chair or between the feet, and throw in any cold article most convenient, as bricks, pieces of iron, &c.

Dr. CRONYN fully agreed in the remarks of Dr. Strong, and had found vapor the most valuable remedy that he had ever used. There is much difference of opinion among writers as to the treatment of croup, but they all believe it to be a diphtheritic disease.

Under five years of age it is almost uniformly fatal. The use of vapor is now *sub judice* by the profession. He has always used it, and last year lost no cases. Generally he gave as remedies, in addition, the iodide of ammonium and the iodate of potash. Saw a case with Dr. Tobie in a family where two children had died under or during treatment. This patient was treated with carbonate of ammonia and vapor, and recovered. He, also, in cases where there is marked diptheritic exudation, gave chlorate of potash and iron.

Dr. WHITE considered the pathology and treatment of croup of great interest, and since he began the practice of medicine there has been a great change in its treatment. He did not think that any particular routine of management are adapted to all cases, but that different cases require diverse treatment, both local and internal. He believed that sustaining remedies were always indicated. He generally combined iron and chlorate potash, as follows: ℞ ferri, muriat., ℥i.; potass. chloratas ℥ss.; sacch. alba., ℥ii.; aq. bulient, Oi. Of this give two teaspoonsful every third hour. Turpeth mineral has been used by Fordyce Barker and others extensively. It is indicated in cases where there is a large accumulation of false membrane, and the patient has sufficient strength. In this class of cases the membrane will often be dislodged by the act of vomiting. The use of vapor is of value in conjunction with the turpeth mineral; but feeble cases always require stimulants. Oxygen has been largely used in New York the past winter for the purpose of keeping the patient alive till the poison can be eliminated from the system. Has seen it prove of great value in some cases. It is also employed in conjunction with vapor medicated with an alkali. In those cases where the obstruction is confined to the larynx, tracheotomy is to be considered. While in Paris learned from Trousseau that the operation is often made; and that about twenty-eight cases in one hundred, or about one in four, recover. When used it should be much earlier than is the practice in this country.

Dr. STRONG is not sure as to the value of the alkali in the vapor, although a solution of bicarbonate of soda will dissolve the false membrane out of the body; but the difficulty, he conceived, lies in getting a sufficient quantity, through the glottis, to be of any value as a solvent. He thinks that the high temperature of the room should not be overlooked. It should be kept at 100° at least.

Influenza, diarrhœa, and malarial fevers, were reported as most prevalent.

Adjourned.

W. C. PHELPS, Secretary.

